# Expression and role of lncRNAs in the regeneration of skeletal muscle following contusion injury

**DOI:** 10.3892/etm.2019.8271

**Published:** 2019-12-02

**Authors:** Lifang Zheng, Xiaoguang Liu, Peijie Chen, Weihua Xiao

Exp Ther Med 18: 2617-2627, 2019; DOI: 10.3892/etm.2019.7871

Subsequently to the publication of this article, the authors have realized that the first pair of authors listed on the paper, Lifang Zheng and Xiaoguang Liu, were not credited as joint first authors who made equal contributions to this study (asterisks should have been included alongside their names with the appropriate footnote to reflect this).

Therefore, the correct author affiliations for this article should have been presented as follows:

LIFANG ZHENG*, XIAOGUANG LIU*, PEIJIE CHEN and WEIHUA XIAO

School of Kinesiology, Shanghai University of Sport, Shanghai 200438, P.R. China

*Contributed equally

The authors regret their oversight in this regard, and apologize for any inconvenience caused.

